# Development of the Pulmonary Embolism Progression (PEP) score for predicting short-term clinical deterioration in intermediate-risk pulmonary embolism: a single-center retrospective study

**DOI:** 10.1007/s11239-024-03051-5

**Published:** 2024-10-22

**Authors:** Jane Ehret, Dorothy Wakefield, Jessica Badlam, Maryellen Antkowiak, Brett Erdreich

**Affiliations:** 1 Department of Medicine, Vassar Brothers Medical Center, 45 Reade Place, Poughkeepsie, NY 12601 USA; 2 Department of Research and Innovation, Vassar Brothers Medical Center, Poughkeepsie, USA; 3 Department of Pulmonary and Critical Care Medicine, University of Vermont Medical Center, Burlington, USA; 4 Department of Pulmonary and Critical Care Medicine, Vassar Brothers Medical Center, Poughkeepsie, USA

**Keywords:** Pulmonary embolism, Risk stratification, Clinical deterioration, Predictive model, PEP score, Prognosis

## Abstract

Accurate risk stratification in acute intermediate-risk pulmonary embolism (PE) is essential. Current prediction scores lack the ability to forecast impending clinical decline. The Pulmonary Embolism Progression (PEP) score aims to predict short-term clinical deterioration (respiratory failure or hemodynamic instability within 72 h) in patients with intermediate-risk PE. This single-center retrospective cohort study analyzed patients with intermediate PE. The outcome of interest was respiratory failure or hemodynamic instability within 72 h. A multivariate logistic regression identified five predictive variables for the final PEP score: use of > 4 L/min of supplemental oxygen above baseline, lactate > 2.0 mmol/L, high-sensitivity cardiac troponin T (hs-cTnT) > 40 ng/L, tricuspid annular plane systolic excursion (TAPSE) < 13 mm, and the combination of central and subsegmental clot. The derivation cohort included 117 patients, and the validation cohort included 70 patients. The area under the receiver operating characteristic (AUROC) curve for the derivation cohort was 0.8671 (95% CI: 0.7946, 0.9292), and for the validation cohort, it was 0.9264 (95% CI: 0.8680, 0.9847). A PEP score of 4 points yielded the highest combination of sensitivity (93%) and specificity (65%). Each incremental point increase in the PEP score raised the probability of clinical deterioration by a factor of 1.933. The PEP score is a reliable tool for predicting the likelihood of clinical deterioration in intermediate-risk PE patients within 72 h, potentially aiding in timely clinical decision-making and improving patient outcomes.

**Graphical abstract:**

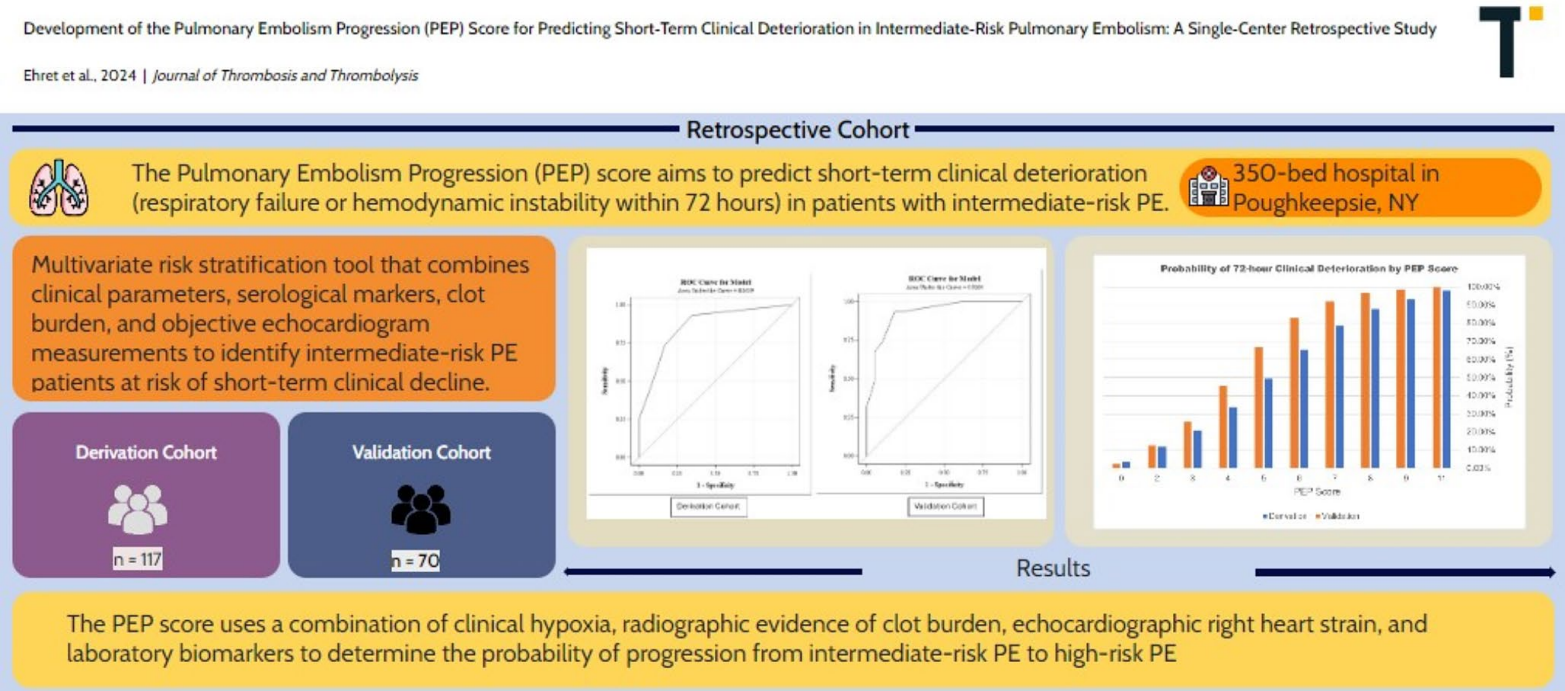

**Supplementary Information:**

The online version contains supplementary material available at 10.1007/s11239-024-03051-5.

## Take home points

Study question: To develop and validate a risk score to determine clinical deterioration of intermediate-risk pulmonary embolism patients.

Results: Using multivariate regression, we identified 5 variables that reliably predict clinical deterioration in patients with intermediate-risk PE.

Interpretation: The PEP score incorporates clinical, laboratory, and radiographic variables to predict which intermediate-risk PE patients will clinically deteriorate within 72 h of diagnosis of pulmonary embolism.

**Supplementary Information:**

The online version contains supplementary material available at 10.1007/s11239-024-03051-5.

Pulmonary embolism (PE) remains a leading cause of mortality worldwide [1]. Early identification of patients with imminent clinical deterioration is crucial to provide timely escalation of care to improve patient outcomes [2].

The 2019 European Society of Cardiology (ESC) guidelines classify patients with acute PE into low, intermediate-low, intermediate-high, and high-risk categories based on hemodynamic stability, evidence of right ventricular dysfunction (RVD), and cardiac biomarkers [3]. This classification system, on the bookends, aids management but lacks predictors of impending clinical deterioration, particularly in the intermediate-risk group. The treatment guidelines for intermediate-risk PE are currently limited, often leaving clinicians to rely on clinical judgment to guide medical management. Furthermore, the diverse spectrum of clinical presentations in this group complicates risk stratification and treatment selection [4, 5].

As of this publication, clinical prediction scores lack the ability to forecast short-term clinical decline in intermediate-risk PE. Scores like the Pulmonary Embolism Severity Index (PESI), simplified PESI (sPESI), and BOVA lack utility for acute management decisions [6–10]. These predictive scores are not validated to identify patients on the brink of cardiac or respiratory collapse. To guide clinical decision making, experts agree that a comprehensive and effective risk assessment model that reflects the complex pathophysiology of acute PE is necessary [10, 11]. The aim of this study focuses on developing and validating a novel prognostic model that predicts short-term clinical deterioration in acute PE. In doing so, we introduce the Pulmonary Embolism Progression (PEP) Score, a quantitative risk stratification tool that combines clinical parameters, serological markers, clot burden, and objective echocardiogram measurements to identify intermediate-risk PE patients at risk of clinical deterioration within 72 h of presentation.

## Study design and methods

A retrospective study took place at a 350-bed community teaching hospital in Poughkeepsie, NY. Patients diagnosed with intermediate PEs between February 2020 and February 2024 underwent a chart review of clinical, laboratory, computed tomography pulmonary angiography (CTPA), and transthoracic echocardiography (TTE) findings from the time of PE diagnosis and for 72 h thereafter. The outcome of interest was short-term clinical deterioration, defined as either respiratory failure or hemodynamic instability, within 72 h of PE diagnosis.

This study was approved by the Institutional Review Board (IRB), with a waiver of informed consent. We adhered to the Transparent Reporting of a Multivariable Prediction Model for Individual Prognosis or Diagnosis (TRIPOD) reporting guideline for reporting multivariate prediction model development and validation [12].

### Patient selection

Subjects were identified through the inpatient discharge database (DAD) using the ICD-10 code for PE. The electronic medical record (EMR) was reviewed to confirm diagnosis of PE by CTPA. All confirmed PE cases were screened for markers of RVD within the first 24 h of admission. Thresholds for RVD included: high-sensitivity cardiac troponin (hs-cTnT) > 30 ng/L, N-terminal pro-brain natriuretic peptide (NT-proBNP) > 300 pg/mL, CTPA findings (RV: LV ratio > 1.0, septal deviation, or retrograde contrast reflux into the vena cava), or TTE findings (RV dilation (end-diastolic diameter > 30 mm), right ventricular systolic pressure (RVSP) > 40 mm Hg, septal deviation, positive McConnell’s sign, or positive “D” sign.

Eligible subjects were ≥ 18 years old with confirmed acute PE and at least one marker of RVD. Low-risk PE patients and pregnant patients were excluded. Subjects were divided into two cohorts. The derivation cohort consisted of all available data at the time of retrieval on December 18, 2023. This cohort was used to develop, evaluate, and internally validate the predictive model and clinical scoring system. The validation cohort consisted of subjects whose data was uploaded after December 19, 2023. This cohort was used to evaluate the model’s performance on an independent sample. The data for both the derivation and validation cohorts was obtained from the same hospital. Subjects missing any predictor variables were excluded from the final derivation and validation models. Subjects were used in a post-hoc extrapolation cohort if missing only one predictor variable.

### Outcome variable

The outcome of interest was whether the subject had clinical deterioration, defined as worsening respiratory failure or hemodynamic instability, within 72 h of PE diagnosis. Respiratory failure was defined as the need for high flow nasal cannula (HFNC), non-invasive positive pressure ventilation (NIPPV), or mechanical ventilation (MV) to maintain oxygen saturation (SpO2) above 90%. Hemodynamic instability was defined as at least one of the following criteria: systolic blood pressure (SBP) less than 90 mm Hg or a drop of greater than 40 mm Hg from baseline for greater than 15 min, the need for catecholamine administration to maintain SBP above 90 mm Hg, or cardiac arrest.

### Data collection

Subjects were collected from a central list, de-identified, and uploaded into a secure Redcap database. Data entry occurred periodically between October 2022 and February 2024. Data was collected from the time of PE diagnosis and for 72 h afterwards. The data collected at the time of PE diagnosis was used for the current study and included the following:

Patient characteristics: Baseline demographics (age, sex, race, BMI); chronic medical conditions (including preexisting heart failure, chronic lung disease, active or past malignancy, and prior venous thromboembolism); additional risk factors for PE (recent COVID-19 or other systemic infection, travel, surgery, or hospitalization).

Clinical data: Symptomatology (syncope, chest pain, dyspnea, leg pain or swelling, altered mental status); highest and lowest heart rate (HR), SBP, and supplemental oxygen (O2) requirements (above baseline) prior to admission. Additional clinical data was collected for 72 h from the time of PE diagnosis, including trends in vital signs, supplemental oxygen requirements, vasopressor requirements (if any), and medical documentation pertaining to escalation of care or clinical deterioration.

Laboratory and imaging results: Venous plasma samples were collected on arrival by laboratory personnel and included hs-cTnT, NT-proBNP, and lactic acid. Radiologists independently reviewed CTPA images and reported the most proximal and distal anatomical thrombus distribution. Radiologists reported the presence or absence of RVD, and the RV: LV ratio as either less than or equal to 1.0, or greater than 1.0. A TTE was performed within 24 h by a certified ultrasound technician and read by in-house cardiologists per the Intersocietal Accreditation Commission (IAC) Standards and Guidelines for Echocardiography Accreditation [13]. The parameters collected included the presence or absence of: RV dilation (end-diastolic diameter greater than or equal to 30 mm from the parasternal view), interventricular septum deviation, clot in transit, McConnell’s sign, and valvular dysfunction. Numerical values were collected for left ventricular ejection fraction (LVEF [%]), RVSP [mm Hg], and TAPSE [mm]. Bilateral lower limb complete duplex ultrasound (CDUS) testing was performed within 24 h by certified ultrasound technicians using two-dimensional imaging, graded compression, and Doppler analysis. Images were reviewed by in-house radiologists and reported as: proximal deep vein thrombosis (DVT), distal DVT, both, or absent.

### Statistical methods

All baseline characteristics and potential predictor variables were compared by study group (Adverse outcome, Y/N). Descriptive data was reported as absolute numbers, percentages, or means +/- standard deviation (means +/- SD) or medians (interquartile range). Subject baseline characteristics and clinical factors, laboratory results, CT, and echocardiogram results were compared by study group [Clinical Deterioration (Yes/No)]. Some continuous variables (TAPSE, Lactate, supplemental O2, hs-cTnT) were converted to dichotomous variables using predefined cut-offs based on clinical significance and prior research studies. Normally distributed continuous variables were compared using the student’s t test, whereas non-normally distributed variables were compared using the Wilcoxon rank-sum tests. Categorical variables were compared using chi-square analysis or Fisher’s exact test. Variables with a significant relationship to the outcome in the univariate analysis were considered as covariates in the multivariable model. A stepwise logistic regression was performed to screen for potential variables for inclusion in the final model. In addition to variables selected by the stepwise procedure, variables deemed clinically relevant by the investigators were included in the selection procedure. Subjects with missing data for significant predictor variables were excluded from analyses by the regression procedure. Five variables associated with an increased risk of short-term clinical deterioration were incorporated into the final regression model. The goodness-of-fit was assessed using Akaike Information Criterion (AIC) and the Hosmer-Lemeshow test. A clinical risk score (PEP Score), ranging from 0 to 11 points, was created based on the results of the final logistic regression model. For each of the five included variables, a point value was assigned based on the rounded square root of the odds ratio. A receiver’s operator curve (ROC) was created to assess the accuracy of the model. For each score cutoff, the probability, sensitivity, specificity, positive predictive value (PPV), and negative predictive value (NPV) were calculated. Youden’s index was calculated to identify the point with the highest sensitivity and specificity. Accuracy was measured using the brier score, measuring the difference between predicted vs. observed outcomes. Using a second dataset, PEP scores were calculated for each subject, and the process was repeated– an ROC curve was created, and the sensitivity, specificity, PPV, NPV were calculated for validation. A post-hoc analysis was performed using the subjects excluded from the derivation and validation cohorts due to missing variables. This modified PEP score (mPEP) included all subjects missing one of the five predictor variables. The missing variable was assumed to be normal if not preset, making the modified PEP score with a reduced potential range of 0 to 9 points. Statistical analysis was performed using the SAS statistical software version 9.4 (SAS Institute, Cary, NC, USA).

## Results

A sample of 244 subjects was used to compare baseline characteristics and clinical factors, laboratory results, CTPA, and TTE results by study group [Clinical deterioration (Y/N)] and identify potential predictors of an adverse outcome.

Altered mental status, complaints of dyspnea, HR > 110, concurrent DVT, RV: LV ratio > 1.0 on CTPA, clot in transit, and McConnell’s sign were independently predictive of the outcome, but lost significance in the multivariate regression analysis. Notable variables that were not significant predictors of 72-hour clinical deterioration included older age, male sex, syncope, chronic lung disease, pre-existing heart failure, and history of malignancy. The results of the univariate analysis are available in the online supplementary material (e-Table 1).

The statistically significant variables were entered into a stepwise logistic regression. The variable selection process resulted in the five predictors of clinical deterioration within 72 h: the use of > 4 L/min of supplemental oxygen (above baseline) to maintain SpO2 > 90% (*p* < 0.01), lactic acid > 2.0 mmol/L (*p* < 0.01), hs-cTnT > 40 ng/L (*p* < 0.01), TAPSE *≤* 13 mm (*p* < 0.01) and the combination of central and subsegmental clot burden (*p* < 0.01).

The derivation cohort (*N* = 117) included subjects with complete data for all five of final variables, of which 45 (38.46%) had clinical deterioration within 72 h. Table 1 displays the baseline patient characteristics of the derivation cohort subjects.

**Table 1:** Baseline characteristics of the derivation cohort

Patient Characteristic	Clinical Deterioration		*P*-value
	No (*N* = 72)	Yes (*N* = 45)	
Age, years, mean (SD)	71.0 (14.4)	63.3 (17.5)	0.01
Sex, female, n (%)	42 (58.3)	21 (46.7)	0.22
Race, n (%)			0.20
Black	13 (18.1)	6 (13.3)	
White	52 (72.2)	31 (68.9)	
Other	5 (6.9)	8 (17.8)	
Unknown	2 (2.8)	0 (0.0)	
BMI, mean (SD)	30.9 (8.4)	33.1 (10.4)	0.22
PESI score, mean (SD)	116.1 (41.2)	134.8 (46.0)	0.02
Pre-existing Conditions			
Chronic lung disease, n (%)	16 (22.2)	11 (24.4)	0.78
Pre-existing heart failure, n (%)	14 (19.4)	8 (17.8)	0.82
Prior VTE, n (%)	14 (19.4)	6 (13.3)	0.39
Active or past malignancy, n (%)	15 (22.4)	11 (25)	0.75
Risk Factors in final model			
Supplemental O2 > 4 L/min above baseline, n (%)	7 (9.7)	22 (48.9)	< 0.01
TAPSE ≤ 13 mm, n (%)	13 (18.1)	22 (48.9)	< 0.01
Central and subsegmental artery thrombus, n (%)	12 (16.7)	18 (40.0)	< 0.01
Hs-cTnT > 40 ng/L, n (%)	34 (47.2)	34 (75.6)	< 0.01
Lactate > 2 mmol/L, n (%)	25 (34.7)	34 (75.6)	< 0.01

BMI = body mass index; SD = standard deviation; Hs-cTnT = high-sensitivity cardiac troponin T; PESI score = pulmonary embolism severity index score; TAPSE = tricuspid annular plane systolic excursion; VTE = venous thromboembolism

The results of the final logistic regression are shown in Table 2. The Hosmer and Lemeshow (HL) (χ² = 5.0273, df = 7, *p* = 0.6566) statistic indicated good fit. The area under the receiver’s operating curve (AUROC) was 0.8671 (SE = 0.0339, 95% CI: 0.8006, 0.9336), indicating good discriminatory ability to predict 72-hour clinical deterioration.

**Table 2:** Logistic regression results and PEP score points

Final predictor variable	Beta (SE)	OR (95% CI)	*P*-value	Points
Supplemental O2 > 4 L/min above baseline	2.15 (0.58)	8.55 (2.74–26.67)	< 0.001	3
Lactate > 2 mmol/L	1.19 (0.52)	3.30 (1.18–9.22)	0.02	2
Hs-cTnT > 40 ng/L	1.22 (0.56)	3.37 (1.14–10.01)	0.03	2
Central + subsegmental thrombus	1.42 (0.59)	4.15 (1.32–13.09)	0.01	2
TAPSE ≤ 13 mm	1.35 (0.56)	3.86 (1.29–11.57)	0.02	2

Beta = beta coefficient; CI = confidence interval; hs-cTnT = high-sensitivity cardiac troponin T; OR = odds ratio; SE = standard deviation; TAPSE = tricuspid annular plane systolic excursion

### PEP score

**Fig. 1:**
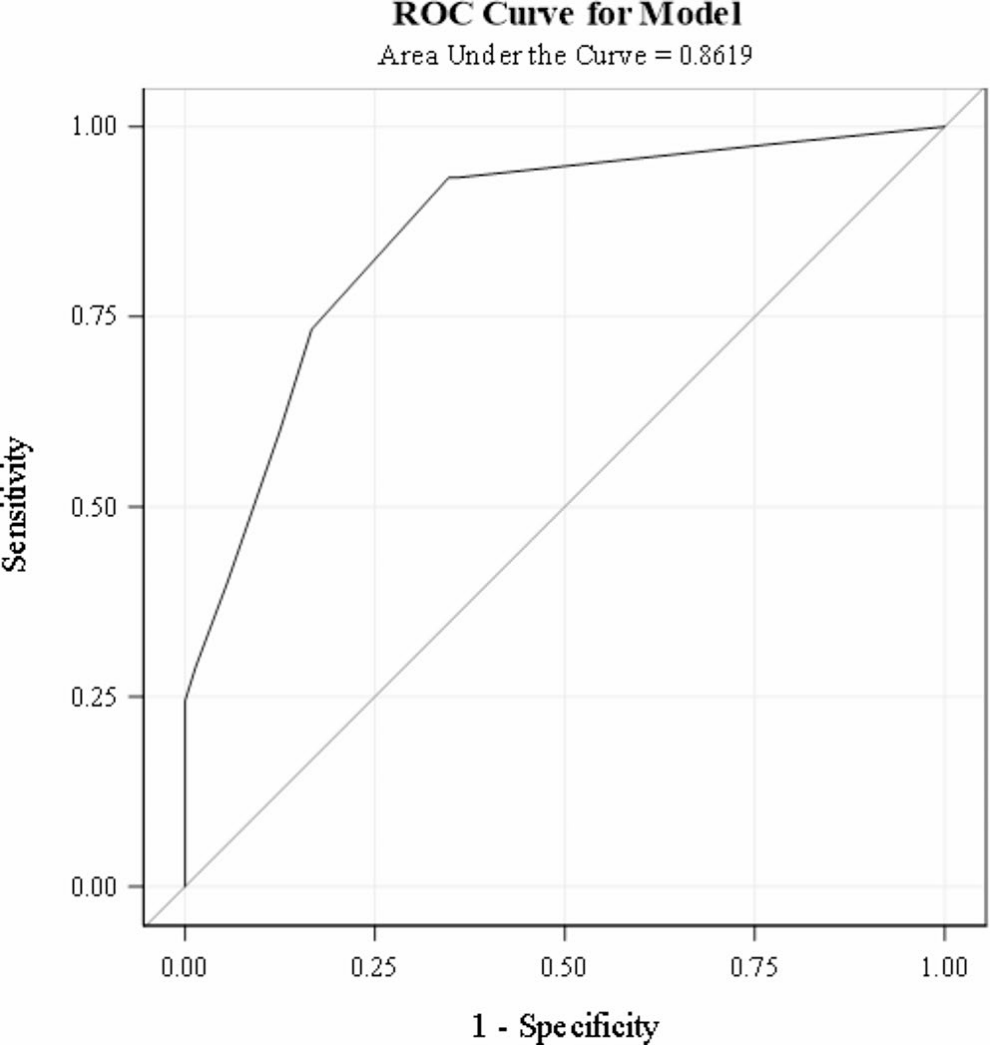
AUROC curve for the derivation cohort

To convert the 5-variable logistic regression model into a clinical score, the rounded square root of the odds ratio (OR) was used to assign a point value to each risk variable and derive the PEP score (Table 2). The odds of clinical deterioration increase by a factor of approximately 1.933 for every one-unit increase in the score. The HL statistic (χ² = 5.4688, df = 5, *p* = 0.3614) supported adequate model fit. The AUROC of PEP Score (Fig. 1) was 0.8619 (SE = 0.0343, 95% CI: 0.7946, 0.9292), showing good discrimination in the points model.

### Validation cohort

The predictive model was validated using a separate cohort. Ninety-two subjects were initially available, but only seventy had full datasets. The outcome was met in 31 (44.29%) of subjects. Compared to the derivation cohort, there were no differences in baseline characteristics. Analysis of the type of clinical decline demonstrated that the validation cohort had fewer subjects with a combination of both respiratory failure and hemodynamic instability compared to the derivation cohort (Table 3).

**Table 3:** Comparison of patient characteristics between derivation and validation cohorts

Patient Characteristic	Derivation Cohort (*n* = 117)	Validation Cohort (*n* = 70)	*P*-value
Clinical Deterioration, n (%)	45 (38.5)	31 (44.3)	0.43
Clinical Deterioration Type, n (%)			0.03
Respiratory Failure	10 (22.2)	5 (16.1)	
Hemodynamic Instability	17 (37.8)	21 (67.7)	
Both	18 (40)	5 (16.1)	
Age, years, mean (SD)	68 (16)	70.2 (15)	0.37
Sex, female, n (%)	63 (53.9)	37 (52.9)	0.90
Race, n (%)			0.27
Black, n (%)	19 (16.2)	7 (10.0)	
White, n (%)	83 (70.9)	58 (82.9)	
Other, n (%)	13 (11.1)	5 (7.1)	
Unknown, n (%)	2 (1.7)	0	
BMI, mean (SD)	31.8 (9.3)	29.6 (8.8)	0.11
PESI score, mean (SD)	123.3 (43.9)	127.6 (52)	0.54
Pre-existing Conditions			
Chronic lung disease, n (%)	27 (23.1)	32 (45.7)	< 0.01
Pre-existing heart failure, n (%)	22 (18.8)	11 (15.7)	0.59
Prior VTE, n (%)	20 (17.1)	13 (18.6)	0.80
Active or past malignancy, n (%)	26 (23.4)	20 (32.3)	0.21
Risk Factors in final model			
Supplemental O2 > 4 L/min above baseline, n (%)	29 (24.8)	20 (28.6)	0.57
TAPSE ≤ 13 mm, n (%)	35 (29.9)	19 (27.1)	0.69
Central and subsegmental artery thrombus, n (%)	30 (25.6)	22 (31.4)	0.39
Hs-cTnT > 40 ng/L, n (%)	68 (58.1)	45 (64.3)	0.40
Lactate > 2 mmol/L, n (%)	59 (50.4)	21 (30)	< 0.01

BMI = body mass index; SD = standard deviation; Hs-cTnT = high-sensitivity cardiac troponin T; PESI score = pulmonary embolism severity index score; TAPSE = tricuspid annular plane systolic excursion; VTE = venous thromboembolism

**Fig. 2:**
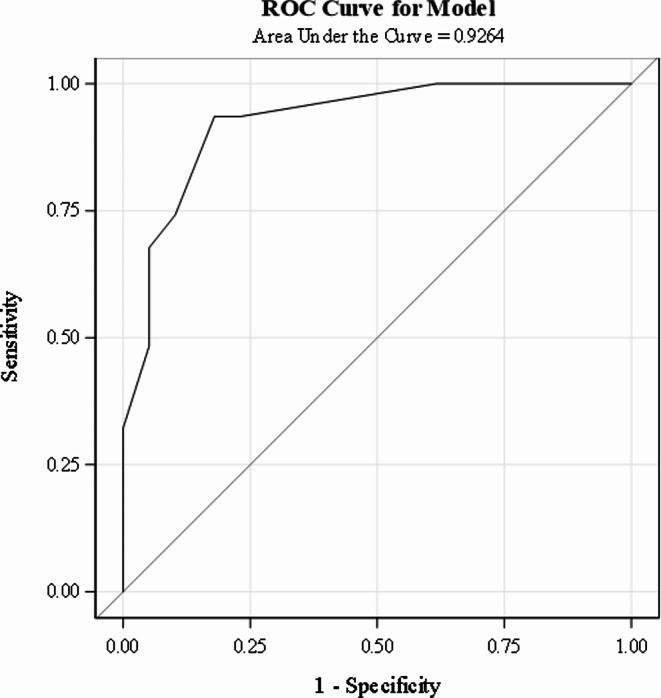
AUROC curve for the validation cohort

PEP scores were calculated using the point system from the derivation cohort (Table 2), and a logistic regression model examined how well the PEP score predicted clinical decline in the validation cohort. The HL statistic (χ² = 5.4693, df = 4, *p* = 0.2424) indicated satisfactory model fit. The AUROC of 0.9264 (SE = 0.0298, 95% CI: 0.8680, 0.9847), indicates good discriminatory ability in a separate cohort of patients (Fig. 2).

Brier scores, ranging from 0 to 1 with lower scores indicating correlation between the predicted and observed outcomes, were computed to further assess calibration and accuracy of the model. Low Brier scores in both the derivation cohort (0.14361) and validation cohort (0.104762) indicated predictive accuracy in each step.

**Fig. 3:**
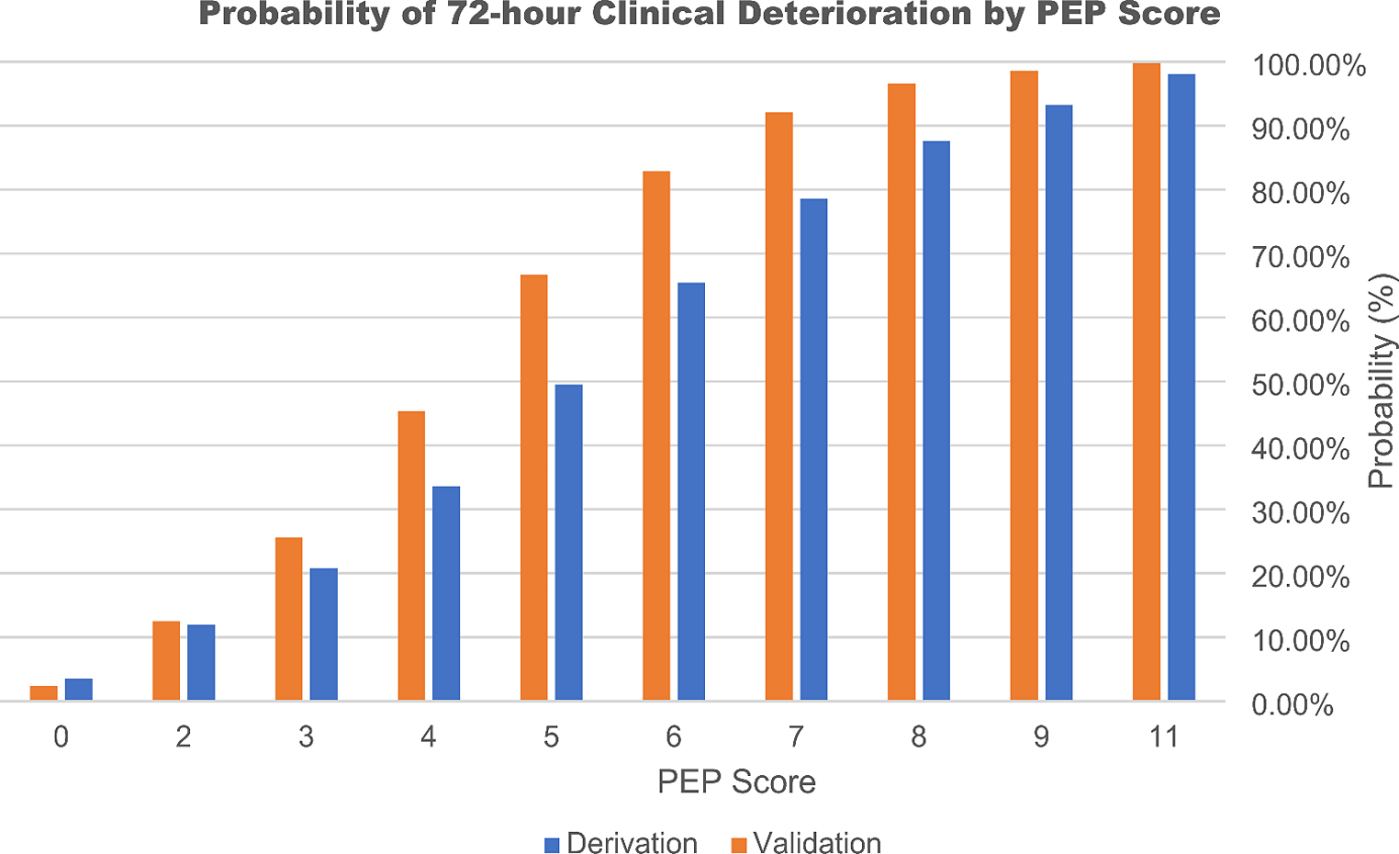
Probability of 72-hour clinical deterioration by PEP score

In both the derivation and validation cohorts, the performance of the PEP score was evaluated across the range of score cutoffs (0–11), yielding different values for probability, sensitivity, specificity, PPV, and NPV (Table 4). Youden’s index indicated that a score of 4 would yield the highest combination of sensitivity (93%) and specificity (65%), with a PPV of 63% and NPV of 94%. As the PEP score increases, both the probability of an adverse outcome and the PPV increase (Table 4). With every one-point increase in the PEP score, the chance of an adverse outcome increases by a factor of approximately 1.933 (Fig. 3). Therefore, although a score of 4 points conveys significant risk of deterioration, a cutoff threshold was not defined to allow for greater clinical applicability.

**Table 4:** Probability, sensitivity, specificity, PPV, and NPV at various PEP score cutoffs. All values are in (%)

	Derivation Cohort PEP Score					Validation Cohort PEP Score				
Cutoff	Probability	Sensitivity	Specificity	PPV	NPV	Probability	Sensitivity	Specificity	PPV	NPV
11	98.08	8.89	100.00	100.00	63.72	99.75	9.68	100.00	100.00	58.21
9	93.19	24.44	100.00	100.00	67.92	98.55	22.58	100.00	100.00	61.90
8	87.63	28.89	98.61	92.86	68.93	96.57	32.26	100.00	100.00	65.00
7	78.55	40.00	94.44	81.82	71.58	92.10	48.39	94.87	88.24	69.81
6	65.45	60.00	87.50	75.00	77.78	82.86	67.74	94.87	91.30	78.72
5	49.49	73.33	83.33	73.33	83.33	66.72	74.19	89.74	85.19	81.40
4	33.63	93.33	65.28	62.69	94.00	45.39	93.55	82.05	80.56	94.12
3	20.77	93.33	63.89	61.76	93.88	25.62	93.55	76.92	76.32	93.75
2	11.94	97.78	22.22	44.00	94.12	12.50	100.00	38.46	56.36	100.00
0	3.50	100.00	0.00	38.46	.	2.40	100.00	0.00	44.29	.

NPV = negative predictive value; PPV = positive predictive value

**Table 5:** Probability, sensitivity, specificity, PPV, and NPV at various modified PEP score cutoffs. All values are in (%)

Modified PEP Score					
Cutoff	Probability	Sensitivity	Specificity	PPV	NPV
9	96.68	18.75	100.00	100.00	90.00
7	80.92	31.25	98.29	71.43	91.27
6	61.83	43.75	97.44	70.00	92.68
5	38.22	75.00	95.73	70.59	96.55
4	19.11	87.50	83.76	42.42	98.00
3	8.27	87.50	79.49	36.84	97.89
2	3.33	100.00	39.32	18.39	100.00
0	0.50	100.00	.	12.03	.

NPV = negative predictive value; PPV = positive predictive value

### Modified PEP score

A post-hoc analysis was performed using subjects excluded from the derivation and validation cohorts for missing one predictor variable (*N* = 133). Clinical deterioration occurred in 16 (12.03%) of these subjects. The missing variables were lactate (*N* = 121), TAPSE (*N* = 10), or hs-cTnT (*N* = 2), all of which are weighted equally in the PEP score. A modified PEP (mPEP) score, using the same point system with a reduced potential range of 0–9 points, was evaluated in this group (Table 5). Missing variables assumed normal.

**Fig. 4:**
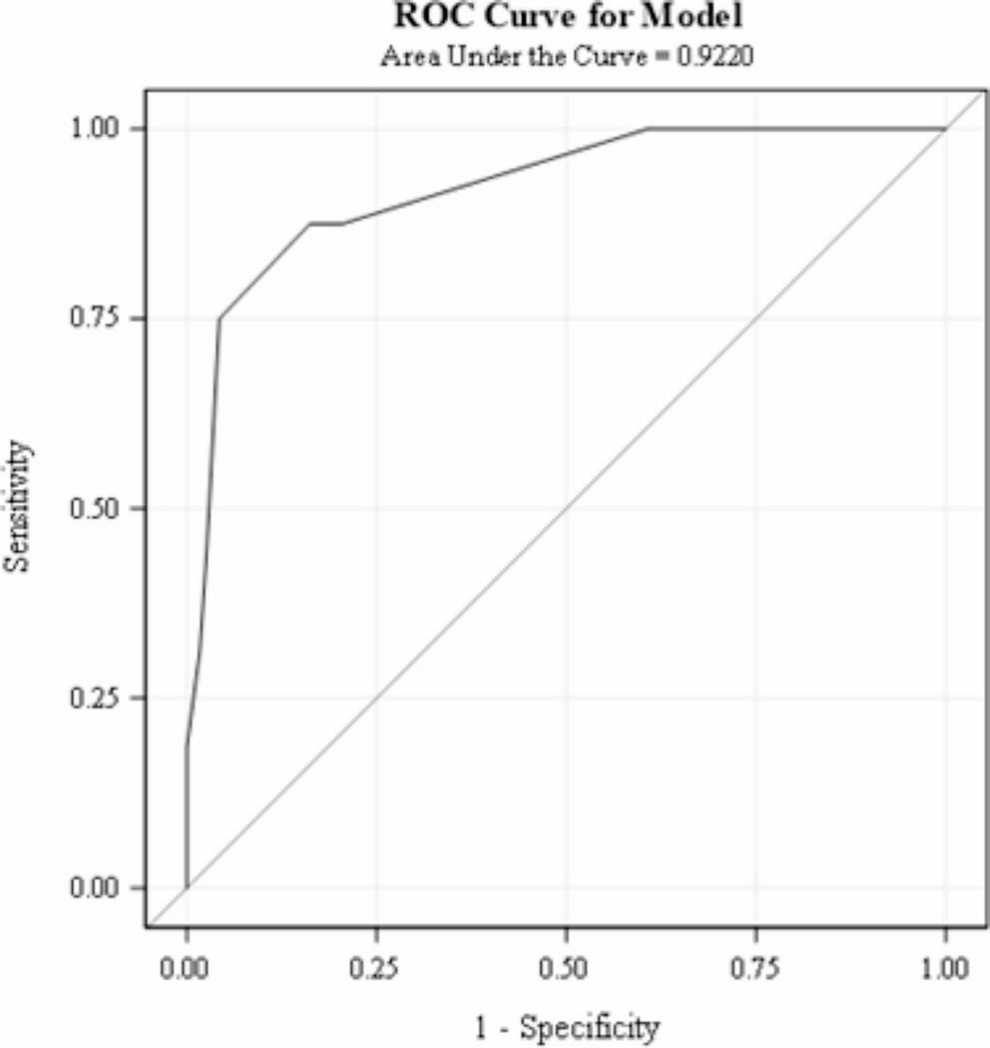
AUROC curve for the mPEP score in the cohort of subjects missing one variable

**Fig. 5:**
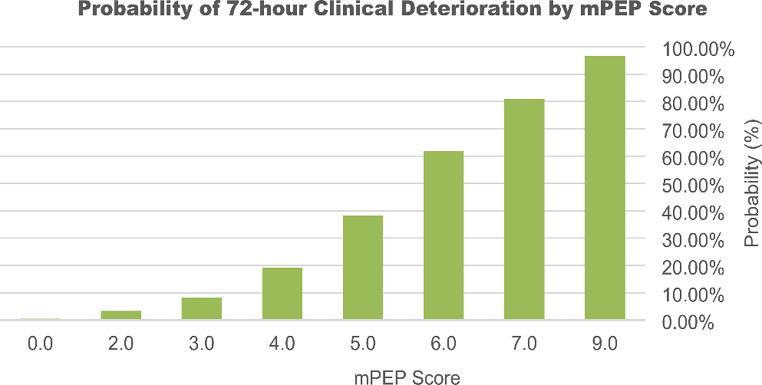
Probability of 72-hour clinical deterioration by mPEP score

The ROC analysis yielded comparable results to the validation cohort, with an AUROC of 0.9220 (SE = 0.0350, 95% CI: 0.835, 0.9906) (Fig. 4). The HL test ((χ² = 1.5109, df = 2, *p* = 0.4698) demonstrates adequate model fit. The low Brier score (0.060425) indicated accuracy in predicting the adverse outcome despite missing one of the variables. Like with the PEP Score, the probability of the adverse outcome increased with higher mPEP scores (Fig. 5).

## Discussion

The present study derives and externally validates a clinical score for predicting short-term deterioration in patients with intermediate-risk PE at a single institution. The PEP score identifies patients at risk of respiratory or hemodynamic collapse within the critical 72-hour of presentation, providing a more immediate and actionable clinical assessment tool for treating clinicians.

The PEP score uses a combination of clinical hypoxia, radiographic evidence of clot burden, echocardiographic RVD, and laboratory biomarkers to determine the probability of progression from intermediate-risk PE to high-risk PE. Interestingly, the clinical degree of hypoxia had the highest weight in our predictive model. Although, in other studies, elevated HR was a predictor of worsening clinical outcomes, our multivariate regression selectively chose hypoxemia as a more clinically significant vital sign [14].

Saddle, main pulmonary artery, lobar artery, and subsegmental artery clots were independently significant in the univariate analysis; however, only subsegmental clot was initially retained through the stepwise regression. We further refined this variable to include central clots as a marker for overall clot burden due to inconsistency in radiologist reporting thrombi as either the most proximal or most distal involved vessels. As previous studies have shown, the percentage of occlusive disease has a significant effect on pulmonary artery pressures and total pulmonary resistance [15, 16]. Further research and modeling on the clinical significance of distal occlusive disease is warranted. Our study suggests that the degree of vascular occlusive disease may be a significant marker for clinical deterioration rather than central clot burden alone [17–19]. Although quantitative markers of clot burden are useful in an academic sense, they are time-consuming without artificial intelligence and have yet to show their usefulness in clinical practice [20].

Decreased RV function, as measured by TAPSE, is independently predictive of short-term mortality in acute PE [21, 22]. Although a value less than 16 mm is considered abnormal, our model shows that 13 mm or less is the best echocardiographic measure for predicting progression to hemodynamic collapse [23]. TAPSE can be obtained quickly at the bedside by a skilled ultrasonographer providing significant amount of diagnostic value. A new marker of RVD uses TAPSE/RVSP to determine the degree of RV-pulmonary artery uncoupling [24, 25]. When comparing our model using TAPSE/RVSP < 0.4 in place of TAPSE < 13 mm, the results were similar. This supports our decision to lower the prognostic threshold of TAPSE from the value of 16 mm that has been used previously.

Lactate serves as a marker of tissue hypoperfusion, even in normotensive PE patients. Elevated lactate is a strong predictor of adverse outcomes in acute PE, especially as it relates to the concept of subclinical shock [26–28]. However, lactate levels were missing most often from initial prognostication. We strongly recommend obtaining lactate levels upon immediate diagnosis of PE with any evidence of right heart strain.

Troponin, but not NT-proBNP, was a predictor of disease progression in our study. Although several studies have shown that higher levels of NT-proBNP at the time of PE diagnosis corelate with increased mortality, its prognostic utility may be limited by patient characteristics such as age, obesity, chronic cardiac conditions, and renal failure [29–31].

Nearly half of the subjects that were collected were excluded from the derivation and validation cohorts due to missing data points. To have the most clinical relevance, we created a modified PEP score that included all subjects that were missing one of the five variables. The modified PEP score was able to predict adverse outcomes despite missing key data points. We hope that moving forward both the PEP score and modified PEP score will have actionable relevance when applied to intermediate-risk patients.

It is worth noting that subjects in this study met our outcome variables whether they had the usual care of systemic anticoagulation or more advanced interventions such as catheter directed interventions. This tool is not intended to recommend a specific treatment modality, and further studies are warranted to determine the appropriate intervention for the intermediate-risk group with high probability of clinical deterioration.

Several limitations of our study should be noted. The retrospective design inherently introduces potential information biases due reliance on existing medical records. Clot location, determined by several radiologists not involved in the study, had inconsistent reporting of either the most proximal and/or distal thrombus. This problem is not inherent to our institution and further work is necessary to standardize CTA reporting. This problem may also be mitigated by adopting artificial intelligence capable of reliably reporting the degree of clot burden and location. Lastly, the PEP score was derived and externally validated at a single institution. Further validation in larger, multicenter cohorts is needed to ensure broader applicability.

## Interpretation

The PEP score helps to determine the risk of clinical deterioration of intermediate-risk PE patients. This tool will provide much needed information to treating clinicians and further research is necessary to determine its benefit on a global scale.

## Electronic supplementary material

Below is the link to the electronic supplementary material.

Supplementary Material 1

## Data Availability

The datasets used during the current stuy are available from the corresponding author on reasonable request.

## Abbreviations

AIC: Akaike Information Criterion
AUROC: Area Under the Receiver Operating Characteristic Curve
BMI: Body Mass Index
BOVA: Bova Score
CI: Confidence Interval
CTPA: Computed Tomography Pulmonary Angiography
DAD: Inpatient Discharge Database
df: Degrees of Freedom
DVT: Deep Vein Thrombosis
EMR: Electronic Medical Record
HFNC: High Flow Nasal Cannula
HL: Hosmer-Lemeshow
HR: Heart Rate
hs-cTnT: High-Sensitivity Cardiac Troponin T
IAC: Intersocietal Accreditation Commission
ICD-10: International Classification of Diseases, Tenth Revision
IRB: Institutional Review Board
LVEF: Left Ventricular Ejection Fraction
mm Hg: Millimeters of Mercury
MV: Mechanical Ventilation
NIPPV: Non-Invasive Positive Pressure Ventilation
NPV: Negative Predictive Value
NT-proBNP: N-Terminal pro-B-type Natriuretic Peptide
OR: Odds Ratio
O2: Oxygen
PE: Pulmonary Embolism
PEP Score: Pulmonary Embolism Progression Score
PPV: Positive Predictive Value
RV: Right Ventricle
RVD: Right Ventricular Dysfunction
RVSP: Right Ventricular Systolic Pressure
SBP: Systolic Blood Pressure
SD: Standard Deviation
SE: Standard Error
SpO2: Oxygen Saturation
TAPSE: Tricuspid Annular Plane Systolic Excursion
TRIPOD: Transparent Reporting of a Multivariable Prediction Model for Individual Prognosis or Diagnosis
TTE: Transthoracic Echocardiography

## References

[1] Jiménez D, de Miguel-Díez J, Guijarro R et al (2016) Trends in the management and outcomes of Acute Pulmonary Embolism: analysis from the RIETE Registry. J Am Coll Cardiol 67(2):162–170. 10.1016/j.jacc.2015.10.060 26791063 10.1016/j.jacc.2015.10.060

[2] Jiménez D, Rodríguez C, León F et al (2022) Randomised controlled trial of a prognostic assessment and management pathway to reduce the length of hospital stay in normotensive patients with acute pulmonary embolism. Eur Respir J 59(2):2100412 Published 2022 Feb 10. 10.1183/13993003.00412-2021 34385269 10.1183/13993003.00412-2021

[3] Konstantinides SV, Meyer G, Becattini C et al (2020) 2019 ESC guidelines for the diagnosis and management of acute pulmonary embolism developed in collaboration with the European Respiratory Society (ERS). Eur Heart J 41(4):543–603. 10.1093/eurheartj/ehz405 31504429 10.1093/eurheartj/ehz405

[4] Weinstein T, Deshwal H, Brosnahan SB (2021) Advanced management of intermediate-high risk pulmonary embolism. Crit Care 25(1):311. 10.1186/s13054-021-03679-2 34461959 10.1186/s13054-021-03679-2 PMC8406617

[5] Leidi A, Bex S, Righini M, Berner A, Grosgurin O, Marti C (2022) Risk stratification in patients with Acute Pulmonary Embolism: current evidence and perspectives. J Clin Med 11(9):2533 Published 2022 Apr 30. 10.3390/jcm11092533 35566658 10.3390/jcm11092533 PMC9104204

[6] Aujesky D, Obrosky DS, Stone RA, Auble TE, Perrier A, Cornuz J, Roy PM, Fine MJ (2005) Derivation and validation of a prognostic model for pulmonary embolism. Am J Respir Crit Care Med 172(8):1041–1046 16020800 10.1164/rccm.200506-862OC PMC2718410

[7] Jiménez D, Aujesky D, Moores L et al (2010) Simplification of the pulmonary embolism severity index for prognostication in patients with acute symptomatic pulmonary embolism. Arch Intern Med 170(15):1383–1389. 10.1001/archinternmed.2010.199 20696966 10.1001/archinternmed.2010.199

[8] Bova C, Sanchez O, Prandoni P et al (2014) Identification of intermediate-risk patients with acute symptomatic pulmonary embolism. Eur Respir J 44(3):694–703. 10.1183/09031936.00006114 24696111 10.1183/09031936.00006114

[9] Fernández C, Bova C, Sanchez O et al (2015) Validation of a model for identification of patients at Intermediate to High Risk for complications Associated with Acute Symptomatic Pulmonary Embolism. Chest 148(1):211–218. 10.1378/chest.14-2551 25633724 10.1378/chest.14-2551

[10] Brunton N, McBane R, Casanegra AI, Houghton DE, Balanescu DV, Ahmad S, Caples S, Motiei A, Henkin S (2024) Risk stratification and management of Intermediate-Risk Acute Pulmonary Embolism. J Clin Med 13(1):257. 10.3390/jcm13010257 38202264 10.3390/jcm13010257 PMC10779572

[11] Jiménez D, Tapson V, Yusen RD et al (2023) Revised paradigm for Acute Pulmonary Embolism Prognostication and Treatment. Am J Respir Crit Care Med 208(5):524–527. 10.1164/rccm.202212-2234VP 37450886 10.1164/rccm.202212-2234VP PMC10492237

[12] Collins GS, Reitsma JB, Altman DG, Moons KG (2015) Transparent reporting of a multivariable prediction model for individual prognosis or diagnosis (TRIPOD): the TRIPOD Statement. Br J Surg 102(3):148–158. 10.1002/bjs.9736 25627261 10.1002/bjs.9736

[13] Intersocietal Accreditation Commission. IAC Standards and Guidelines for Echocardiography Accreditation. Intersocietal Accreditation Commission. Published 2024. Accessed June 15 (2024) https://www.intersocietal.org/echo/standards/IAC_Standards_and_Guidelines_for_Echocardiography_Accreditation.pdf

[14] Jaureguízar A, Jiménez D, Bikdeli B et al (2022) Heart rate and mortality in patients with Acute Symptomatic Pulmonary Embolism. Chest 161(2):524–534. 10.1016/j.chest.2021.08.059 34478718 10.1016/j.chest.2021.08.059

[15] Azarian R, Wartski M, Collignon MA et al (1997) Lung perfusion scans and hemodynamics in acute and chronic pulmonary embolism. J Nucl Med 38(6):980–983 9189155

[16] Irmak I, Sertçelik Ü, Öncel A et al (2020) Correlation of thrombosed vessel location and clot burden score with severity of disease and risk stratification in patients with acute pulmonary embolism. Anatol J Cardiol 24(4):247–253. 10.14744/AnatolJCardiol.2020.55013 33001050 10.14744/AnatolJCardiol.2020.55013 PMC7585957

[17] Çildag MB, Gok M, Karaman CZ (2017) Pulmonary artery obstruction index and Right Ventricular Dysfunction Signs in Initial and follow up pulmonary computed tomography angiography in Acute Pulmonary Embolism. J Clin Diagn Res 11(7):TC21–TC25. 10.7860/JCDR/2017/28740.10296 28893001 10.7860/JCDR/2017/28740.10296 PMC5583862

[18] Xi L, Xu F, Kang H et al (2024) Clot ratio, new clot burden score with deep learning, correlates with the risk stratification of patients with acute pulmonary embolism. Quant Imaging Med Surg 14(1):86–97. 10.21037/qims-23-322 38223063 10.21037/qims-23-322 PMC10784004

[19] Higazi MM, Fattah RARA, Abdelghany EA, Ghany HSA (2020) Efficacy of computed Tomography Pulmonary Angiography as non-invasive imaging biomarker for risk stratification of Acute Pulmonary Embolism. J Clin Imaging Sci 10:49 Published 2020 Aug 17. 10.25259/JCIS_75_2020 32874754 10.25259/JCIS_75_2020 PMC7451145

[20] Liu W, Liu M, Guo X et al (2020) Evaluation of acute pulmonary embolism and clot burden on CTPA with deep learning. Eur Radiol 30(6):3567–3575. 10.1007/s00330-020-06699-8 32064559 10.1007/s00330-020-06699-8

[21] Pruszczyk P, Goliszek S, Lichodziejewska B et al (2014) Prognostic value of echocardiography in normotensive patients with acute pulmonary embolism. JACC Cardiovasc Imaging 7(6):553–560. 10.1016/j.jcmg.2013.11.004 24412192 10.1016/j.jcmg.2013.11.004

[22] Paczyńska M, Sobieraj P, Burzyński Ł et al (2016) Tricuspid annulus plane systolic excursion (TAPSE) has superior predictive value compared to right ventricular to left ventricular ratio in normotensive patients with acute pulmonary embolism. Arch Med Sci 12(5):1008–1014. 10.5114/aoms.2016.57678 27695491 10.5114/aoms.2016.57678 PMC5016574

[23] Rudski LG, Lai WW, Afilalo J et al (2010) Guidelines for the echocardiographic assessment of the right heart in adults: a report from the American Society of Echocardiography endorsed by the European Association of Echocardiography, a registered branch of the European Society of Cardiology, and the Canadian Society of Echocardiography. J Am Soc Echocardiogr 23(7):685–788. 10.1016/j.echo.2010.05.010 20620859 10.1016/j.echo.2010.05.010

[24] Lyhne MD, Kabrhel C, Giordano N et al (2021) The echocardiographic ratio tricuspid annular plane systolic excursion/pulmonary arterial systolic pressure predicts short-term adverse outcomes in acute pulmonary embolism. Eur Heart J Cardiovasc Imaging 22(3):285–294. 10.1093/ehjci/jeaa243 33026070 10.1093/ehjci/jeaa243

[25] He Q, Lin Y, Zhu Y et al (2023) Clinical Usefulness of Right Ventricle-Pulmonary Artery Coupling in Cardiovascular Disease. *J Clin Med*.;12(7):2526. Published 2023 Mar 27. 10.3390/jcm12072526 10.3390/jcm12072526 PMC10095537 37048609

[26] Vanni S, Jiménez D, Nazerian P et al (2015) Short-term clinical outcome of normotensive patients with acute PE and high plasma lactate. Thorax 70(4):333–338. 10.1136/thoraxjnl-2014-206300 25661114 10.1136/thoraxjnl-2014-206300

[27] Wang Y, Feng Y, Yang X, Mao H (2022) Prognostic role of elevated lactate in acute pulmonary embolism: a systematic review and meta-analysis. Phlebology 37(5):338–347. 10.1177/02683555221081818 35282737 10.1177/02683555221081818

[28] Ebner M, Pagel CF, Sentler C et al (2021) Venous lactate improves the prediction of in-hospital adverse outcomes in normotensive pulmonary embolism. Eur J Intern Med 86:25–31. 10.1016/j.ejim.2021.01.021 33558162 10.1016/j.ejim.2021.01.021

[29] Redfield MM, Rodeheffer RJ, Jacobsen SJ, Mahoney DW, Bailey KR, Burnett JC Jr (2002) Plasma brain natriuretic peptide concentration: impact of age and gender. J Am Coll Cardiol 40(5):976–982. 10.1016/s0735-1097(02)02059-4 12225726 10.1016/s0735-1097(02)02059-4

[30] Krauser DG, Lloyd-Jones DM, Chae CU et al (2005) Effect of body mass index on natriuretic peptide levels in patients with acute congestive heart failure: a ProBNP Investigation of Dyspnea in the Emergency Department (PRIDE) substudy. Am Heart J 149(4):744–750. 10.1016/j.ahj.2004.07.010 15990762 10.1016/j.ahj.2004.07.010

[31] Ezekowitz JA, Alemayehu W, Rathwell S et al (2022) The influence of comorbidities on achieving an N-terminal pro-b-type natriuretic peptide target: a secondary analysis of the GUIDE-IT trial. ESC Heart Fail 9(1):77–86. 10.1002/ehf2.1369 34784657 10.1002/ehf2.13692 PMC8787989

