# Supplementary material for: Development of the Pulmonary Embolism Progression (PEP) score for predicting short-term clinical deterioration in intermediate-risk pulmonary embolism: a single-center retrospective study
Source: J Thromb Thrombolysis. 2024 Oct 22;58(2):243–53. doi: 10.1007/s11239-024-03051-5 (PMC11885318; doi:10.1007/s11239-024-03051-5)
Supplement: Supplementary file 1 — Supplementary Material 1 [file 11239_2024_3051_MOESM1_ESM.docx]

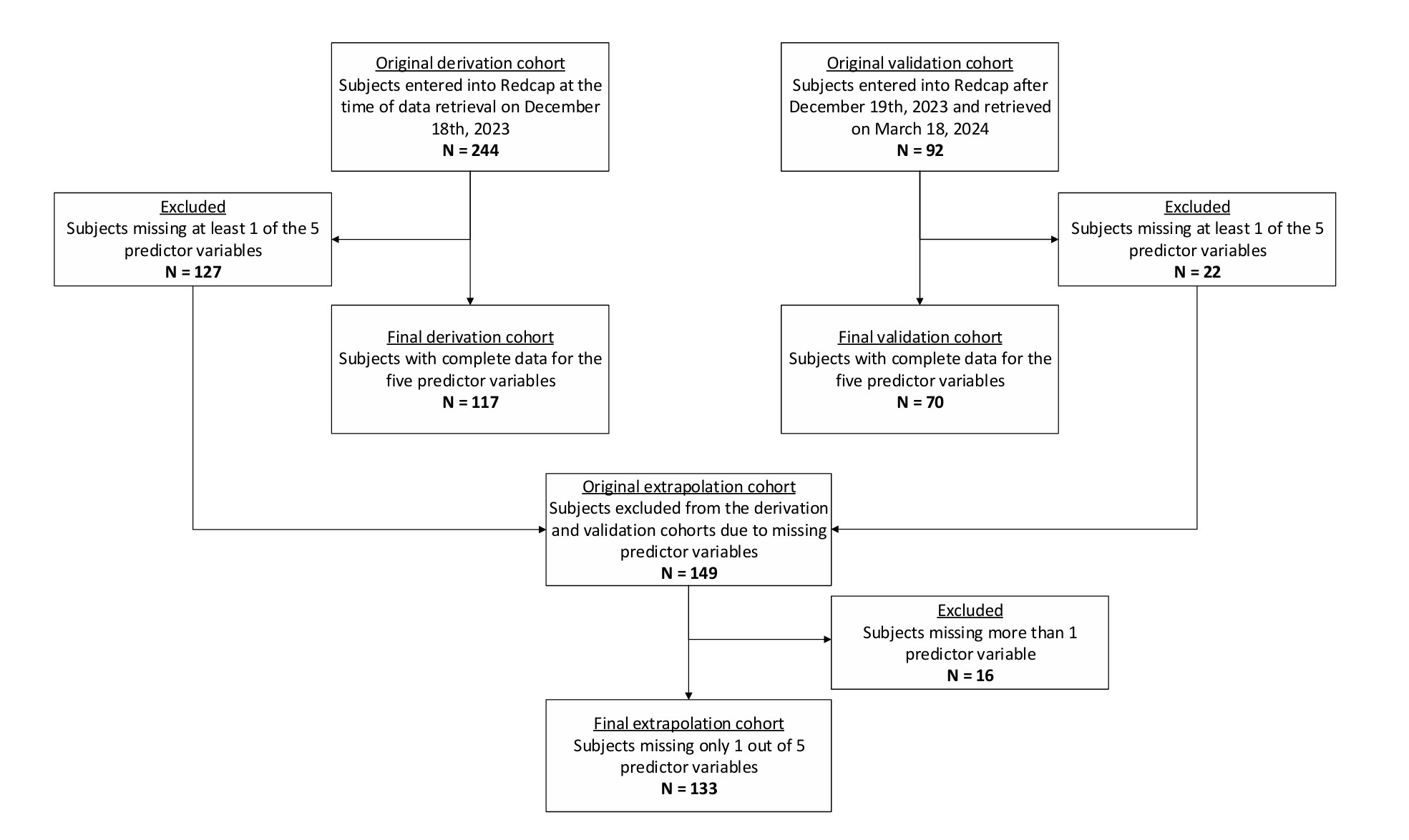


**e-Figure 1: STROBE diagram of the inclusion and exclusion of subjects in the derivation, validation, and extrapolation cohorts.**
The final derivation cohort was used to develop the PEP score. The validation cohort was used to test the PEP score on an independent sample. The extrapolation cohort was used to test the PEP score on subjects missing 1 of the 5 predictor variables, referred to as the modified PEP score.

|  |  |  |  |
| --- | --- | --- | --- |
|  | Clinical Deterioration within 72 hours | |  |
| Clinical/Historical Variables, N (%) | No (N=192) | Yes (N=52) | P-value |
| HR > 110 bpm | 69 (35.9) | 31 (59.6) | 0.002 |
| Supplemental O2 > 4L/min (above baseline)^1^ | 18 (9.4) | 28 (53.9) | <0.001 |
| Dyspnea | 150 (78.1) | 47 (90.4) | 0.05 |
| Altered mental status | 15 (7.8) | 11 (21.2) | 0.005 |
| Syncope | 21 (10.9) | 10 (19.2) | 0.11 |
| Presence of DVT | 122 (63.5) | 40 (76.9) | 0.07 |
| Active/past malignancy | 42 (23.5) | 13 (25.5) | 0.76 |
| Prior PE or DVT | 39 (20.3) | 7 (13.5) | 0.26 |
| Pre-existing heart failure | 25 (13.0) | 8 (15.4) | 0.66 |
| Chronic lung disease | 50 (26.0) | 12 (23.1) | 0.66 |
| Recent systemic infection | 24 (12.5) | 14 (26.9) | 0.01 |
| Echocardiogram Findings, N (%) |  |  |  |
| RV dilation | 77 (40.1) | 42 (80.8) | <0.001 |
| Septal flattening^2^ | 49 (25.5) | 39 (75) | <0.001 |
| Clot in transit | 5 (2.6) | 8 (15.4) | <0.001 |
| McConnell sign | 27 (14.1) | 21 (40.4) | <0.001 |
| RVSP > 40 mm Hg | 72 (48.0) | 32 (72.7) | <0.001 |
| TAPSE ≤ 13 mm | 20 (11.3) | 22 (43.1) | <0.001 |
| CTPA Findings, N (%) |  |  |  |
| RV:LV ratio > 1.0 | 98 (53.0) | 42 (84.0) | <0.001 |
| Saddle thrombus | 35 (18.23) | 25 (48.1) | <0.001 |
| Left main artery thrombus | 87 (45.3) | 35 (67.3) | 0.004 |
| Right main artery thrombus | 98 (51.0) | 41 (78.9) | <0.001 |
| Left lobar artery thrombus | 91 (47.4) | 34 (65.4) | 0.02 |
| Right lobar artery thrombus | 104 (54.2) | 39 (75.0) | 0.007 |
| Left segmental artery thrombus | 78 (40.6) | 29 (55.8) | 0.05 |
| Right segmental artery thrombus | 92 (48.0) | 30 (57.7) | 0.21 |
| Left subsegmental artery thrombus | 32 (16.7) | 18 (34.7) | 0.004 |
| Right subsegmental artery thrombus | 35 (18.2) | 20 (38.5) | 0.002 |
| Right and/or left subsegmental artery thrombus | 42 (21.9) | 22 (42.3) | <0.01 |
| Central (saddle or main or lobar artery) thrombus | 147 (76.6) | 47 (90.4) | 0.03 |
| Central and subsegmental artery thrombus^3^ | 27 (14.1) | 20 (38.5) | <0.001 |
| Laboratory Results, N (%) |  |  |  |
| Hs-cTnT T > 40 ng/L | 89 (47.9) | 39 (76.5) | <0.001 |
| Lactate > 2 mmol/L | 25 (32.1) | 35 (76.1) | <0.001 |
| NT-proBNP > 300 pg/mL | 102 (53.1) | 39 (75.0) | 0.06 |

**e-Table 1: Univariate analysis of the original derivation cohort.**Chi-Square test was used to compare variables between those who had clinical deterioration and those who did not. The original derivation cohort included 244 subjects before excluding those with missing predictor variables.
DVT = Deep Vein Thrombosis; HR = Heart Rate, Hs-cTnT = High-sensitivity Cardiac Troponin T; NT-proBNP: N-Terminal pro-B-type Natriuretic Peptide; PE = pulmonary embolism; RVSP = Right Ventricular Systolic Pressure; TAPSE = Tricuspid Annular Plane Systolic Excursion

1. Supplemental oxygen requirements, rather than SpO2, was used as a marker of hypoxemia because many patients brought to the emergency department by ambulance were already on supplemental O2.
2. Septal flattening was in the initial mode, but it was replaced with TAPSE < 13 mm to improve clinical objectivity.
3. Central thrombus, defined as clot at the pulmonary trunk bifurcation (“saddle”) and/or in the main or lobar pulmonary arteries, was combined with subsegmental thrombus to serve as a surrogate for clot burden.
